# Perspectives on Orthology During the Quest for Orthologs

**DOI:** 10.1007/s00239-025-10290-4

**Published:** 2025-11-20

**Authors:** Natasha Glover, David A. Liberles

**Affiliations:** 1 SIB Swiss Institute of Bioinformatics, 1015 Lausanne, Switzerland; 2 Department of Computational Biology, University of Lausanne, 1015 Lausanne, Switzerland; 3 Department of Biology and Center for Computational Genetics and Genomics, Temple University, Philadelphia, PA 19122 USA

**Keywords:** Orthology, Gene duplication, Gene function, Machine learning, Protein domains, Phylogenetic analysis

## Abstract

This special issue from the Quest for Orthologs community highlights ongoing challenges in detecting and characterizing orthologous genes in the annotation of protein functions. Eleven articles are presented describing ongoing research in this area.

The continued expansion of genome sequencing across the tree of life has transformed evolutionary biology and comparative genomics, leading to a more fundamental understanding of genomic biology. Projects such as the Earth BioGenome Project (Lewin et al. 2018) and numerous clade-specific initiatives are producing vast numbers of annotated genomes. As genome sequencing expands across the tree of life, understanding the biochemical and biological functions of genes encoded in genomes is critical to understanding the genomes themselves. This wealth of data in genome sequences offers unprecedented opportunities to understand gene and genome evolution, yet it also raises substantial challenges for orthology inference. The identification of orthologs, genes that diverged through speciation, remains a cornerstone of comparative analyses, providing the basis for functional annotation transfer, phylogenetic reconstruction, and the study of gene family evolution.

This special issue of the *Journal of Molecular Evolution* grew out of discussions at the 8th Quest for Orthologs (QfO) meeting, held in Montréal, Quebec, Canada in July, 2024. The meeting brought together method developers and researchers who use orthology to examine how orthology inference can adapt to the rapidly changing landscape of genomics. The eleven papers collected here span conceptual re-evaluations, methodological innovations, and practical tools. Taken together, they provide a snapshot of a field that is dynamic, constantly improving, and increasingly integrative. A full report of the meeting is presented in this issue by Majidian et al. (2025).

## Revisiting the Ortholog Conjecture and Functional Divergence

The ortholog conjecture, the notion that orthologs retain functions more consistently than paralogs, has long guided functional annotation. Several contributions in this issue revisit and refine this central idea.

Langschied et al. (2025) argue that orthologs are not immune to functional divergence and that functional equivalence should be treated as a hypothesis to be explicitly tested rather than assumed. They emphasize the importance of assessing both biochemical activity and broader functional context, such as interaction partners (e.g. shared pathways and complexes), when transferring annotations across species. In a complementary perspective, Perez et al. (2025) frames gene function in quantitative biochemical terms. By considering changes in evolutionary processes, including selective optima and strength, their work highlights how functional shifts can occur independently of sequence similarity. This biochemical framing provides a rigorous foundation for modeling functional divergence of all homologs.

Shaw et al. (Shaw et al. 2025) examine the potential of protein language models (PLMs) to probe the ortholog conjecture. They demonstrate that embeddings capture functional constraints across deep evolutionary distances, but also caution that PLMs inherit biases from training data and are less effective at distinguishing near-neutral changes at short timescales. They propose the use of embedding-tree versus gene-tree comparisons as a means of detecting unexpected functional divergence within gene families.

Together, these contributions highlight a nuanced view of the ortholog conjecture: orthologs often, but not always, retain function, and computational methods for transferring functional annotation must account for both gradual and abrupt divergence.

## Broadening the Framework of Orthology

While orthology is often considered at the gene level, several papers in this issue demonstrate the importance of extending the framework to other evolutionary units.

Domains have been a long-standing topic in the orthology community, but we have only scratched the surface of understanding their effect and importance on both genome evolution and orthology inference. In order to investigate the evolution of domains in Metazoa, Xiao et al. (2025) applied a birth–death–gain model to protein domains across metazoans and their unicellular relatives. Their analyses reveal contrasting evolutionary modes: expansion, remodeling, specialization, and streamlining, and they demonstrated that domain repertoires evolve dynamically and often in concert across pathways, in presenting ancestral histories for domain families. Domains with similar functions tend to show similar domain dynamic rate profiles. They underscore the role of domain rearrangements in altering protein function.

As emphasized in the QfO meeting report by Majidian et al. (2025), orthology is increasingly being extended beyond the gene level. Several contributions highlighted the importance of domains as evolutionary units with distinct gain-loss dynamics, while others argued for frameworks that incorporate transcript isoforms generated by alternative splicing. In addition, the report underscored emerging efforts to define orthology for non-coding RNAs, particularly microRNAs. These perspectives reinforce the view that orthology should not be confined to whole genes, but should flexibly accommodate the multiple levels at which genomes encode function.

These studies converge on the conclusion that orthology cannot be defined at the gene level alone. Domains, isoforms, and lineage-specific patterns all contribute to functional diversification and must be integrated into future frameworks. As highlighted by Sarton-Lohéac et al. (2025), hierarchical orthologous groups (HOGs) provide a coherent evolutionary framework that unifies gene, domain, and family-level relationships across taxonomic depths. By explicitly representing duplication and loss events within a phylogenetic context, HOGs exemplify how orthology and paralogy can be structured beyond pairwise gene relationships, enabling integrated analyses of genome evolution, function, and ancestral organization.

## Methodological Developments and Practical Tools

A recurring emphasis of this issue is the need for robust, accessible tools to work with orthology data at scale. Several papers in this special issue address that.

Kharrazi et al. (2025) introduce *OrthoXML-tools*, a suite of tools for parsing, manipulating, and converting hierarchical orthology data. Their contribution is aimed at researchers who wish to analyze or integrate orthology results from different sources. Schoenstein et al. (2025) present *Profylo*, a python package that brings together multiple phylogenetic profiling approaches. It provides unified similarity metrics, clustering algorithms, and visualization features, and facilitates systematic detection of co-evolving genes and benchmarking across methods. Williams and Thomas (2025) describe *OrthoGrafter*, which enables new query sequences to be grafted onto curated reconciled gene trees in the PANTHER database. This tool provides methodology for researchers to integrate their data with an established orthology framework, without performing full orthology inference. Additionally, Swenson et al. (2025) formalize the concept of hierarchical synteny, introducing algorithms to define and rigorously compare syntenic blocks. Their approach allows synteny to be incorporated into orthology inference in a principled way, extending beyond the informal use common in many pipelines.

The NCBI Orthologs resource (Oh et al. 2025) further strengthens this ecosystem by introducing a scalable, high-precision framework for orthology inference within the RefSeq environment. By integrating protein similarity, nucleotide-level conservation, and microsynteny, it delivers one-to-one ortholog assignments across eukaryotes and ensures consistent cross-referencing between RefSeq, Gene, and other NCBI resources.

Together, these tools illustrate the growing interconnectedness of the orthology community. They not only enhance interoperability between databases and methods but also lower barriers for users seeking to perform large-scale comparative analyses. Accessible, well-documented, and benchmarked resources are vital for translating orthology inference from specialized research into broadly applicable infrastructure that underpins functional genomics, evolutionary biology, and genome annotation.

## Conclusions and Outlook

The eleven papers in this special issue collectively advance the theory and practice of orthology. By revisiting the ortholog conjecture, they highlight the importance of testing functional equivalence and accounting for divergence. By expanding the framework to include domains, isoforms, and lineage-specific patterns, they underscore the multidimensional nature of genome evolution. By introducing new tools for phylogenetic profiling, OrthoXML-handling, grafting of sequences to phylogenetic trees, and synteny, they provide new solutions for researchers navigating large genomic datasets. Complementary perspectives on one-to-one versus broader orthology, show that inference must be adapted to context and purpose.

Several common themes emerge. First, benchmarking remains a central challenge, particularly for hierarchical frameworks such as HOGs. Second, functional annotation transfer requires integration of multiple lines of evidence, from biochemistry to embeddings to genomic context. Third, orthology is not only a methodological problem but also a community endeavor, where progress depends on the interoperability of resources, the openness of tools, and the continued dialogue between method developers and users.

In summary, this issue represents both a state-of-the-art synthesis and a call for further work. As genomic data continue to accumulate, orthology detection will remain indispensable, but only if our understanding of what it means evolves in tandem with the biological questions it seeks to address.
